# Phenoxybenzamine Is Neuroprotective in a Rat Model of Severe Traumatic Brain Injury

**DOI:** 10.3390/ijms15011402

**Published:** 2014-01-20

**Authors:** Thomas F. Rau, Aakriti Kothiwal, Annela Rova, Joseph F. Rhoderick, David J. Poulsen

**Affiliations:** Department of Biomedical and Pharmaceutical Sciences, University of Montana, Missoula, MT 59812, USA; E-Mails: Thomas.Rau@mso.umt.edu (T.F.R.); aakriti_kothiwal@yahoo.com (A.K.); annela.rova@umconnect.umt.edu (A.R.); fred.rhoderick@mso.umt.edu (J.F.R.)

**Keywords:** phenoxybenzamine, traumatic brain injury, neuroprotection, morris water maze

## Abstract

Phenoxybenzamine (PBZ) is an FDA approved α-1 adrenergic receptor antagonist that is currently used to treat symptoms of pheochromocytoma. However, it has not been studied as a neuroprotective agent for traumatic brain injury (TBI). While screening neuroprotective candidates, we found that phenoxybenzamine reduced neuronal death in rat hippocampal slice cultures following exposure to oxygen glucose deprivation (OGD). Using this system, we found that phenoxybenzamine reduced neuronal death over a broad dose range (0.1 μM–1 mM) and provided efficacy when delivered up to 16 h post-OGD. We further tested phenoxybenzamine in the rat lateral fluid percussion model of TBI. When administered 8 h after TBI, phenoxybenzamine improved neurological severity scoring and foot fault assessments. At 25 days post injury, phenoxybenzamine treated TBI animals also showed a significant improvement in both learning and memory compared to saline treated controls. We further examined gene expression changes within the cortex following TBI. At 32 h post-TBI phenoxybenzamine treated animals had significantly lower expression of pro-inflammatory signaling proteins CCL2, IL1β, and MyD88, suggesting that phenoxybenzamine may exert a neuroprotective effect by reducing neuroinflammation after TBI. These data suggest that phenonxybenzamine may have application in the treatment of TBI.

## 1. Introduction

Traumatic brain injury (TBI) is a national health concern that affects over 1.7 million individuals each year in the United States [1–3]. Of further concern is the lack of effective treatments to reduce the primary or secondary phase of neuropathology induced by TBI. The development of novel neuroprotective agents has proven difficult as TBI represents a heterologous injury [4–9]. In an effort to discover new treatments for TBI, we have used a rat organotypic, hippocampal slice culture model of oxygen glucose deprivation (OGD) as an initial screen to identify potential neuroprotective candidate compounds [10]. While it is clear there are differences between stroke and TBI, there are similarities in the mechanisms that lead to neuropathology. Both injuries induce the development of inflammation, reactive oxygen species (ROS), reactive nitrogen species (RNS), excitotoxicity, calcium dysregulation, and apoptosis [5,8–12]. TBI also results in sheared blood vessels leading to impaired blood flow and ischemia [11–13]. Thus, utilizing an *in vitro* model of OGD to screen for potential neuroprotective compounds represents an efficient method to identify potential neuroprotective compounds. We previously used this approach to identify low dose methamphetamine as a potential neuroprotective candidate, which was highly effective when tested in the rat lateral fluid percussion (LFP) injury model of severe TBI [10].

Using the rat hippocampal slice culture model, we have recently made the novel observation that phenoxybenzamine exerts a profound neuroprotective effect following OGD. Currently, phenoxybenzamine is an FDA approved treatment for humans and animals to reduce hypertension and excessive sweating associated with adrenal tumors (pheochromocytoma) [14–16]. Pharmacologically, phenoxybenzamine (Dibenzyline; Wellspring Pharmaceuticals) is a halo-alkylamine that blocks both α-1 and α-2 adrenergic receptors, but has a higher affinity for the α-1 receptor [16,17]. Following i.v. administration, receptor antagonism achieves a peak effect at approximately 1 h [17]. However, phenoxybenzamine exerts a long-term effect in the brain with a half-life of approximately 24 hours. As an antagonist, phenoxybenzamine forms an irreversible covalent bond with α-adrenergic receptors, thus the effect of the drug lasts longer than its metabolic half-life [17]. This observation is supported by studies showing the reversal of the effect of phenoxybenzamine is dependent on the synthesis of new receptors and not metabolism of the drug. Hamilton *et al.* demonstrated that only 50% of α-1 receptors had recovered eight days after a single administration of phenoxybenzamine [17]. Based on prior FDA approval of phenoxybenzamine, its stability and ease of administration, relatively long effect, and the absence of harmful side effects; we elected to further investigate phenoxybenzamine as a potential neuroprotective agent in the treatment of TBI.

As a proof-of-concept study, we utilized the rat LFP injury model to test the neuroprotective potential of phenoxybenzamine [18]. Importantly, we chose the clinically relevant time point of 8 h after injury to test the potential neuroprotective effect. The administration of a single, i.v., bolus dose of 1.0 mg/kg at 8 h after injury resulted in significant, long-term behavioral and cognitive improvements. To investigate the possible mechanisms of phenoxybenzamine-mediated neuroprotection we performed a gene array analysis and identified relevant gene targets that were altered as a result of phenoxybenzamine treatment and then confirmed the changes in gene expression by quantitative real-time PCR.

## 2. Results and Discussion

### 2.1. Phenoxybenzamine Prevents Hippocampal Cell Death after Oxygen Glucose Deprivation

**Figure 1.**
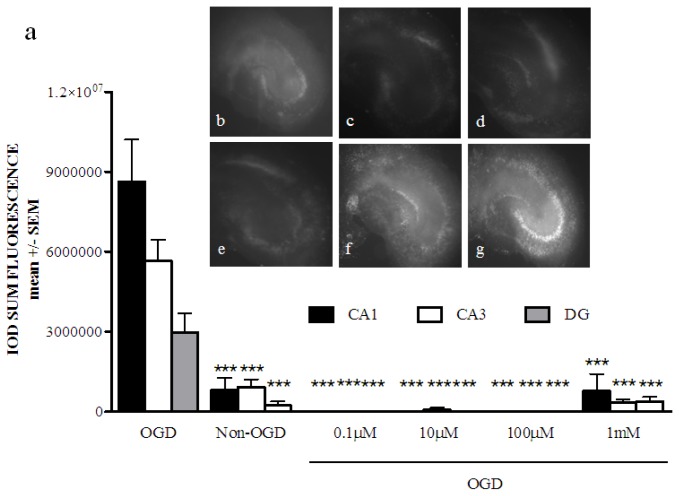
(**a**) Phenoxybenzamine, over a broad dose range, provides significant neuroprotection from oxygen glucose deprivation in rat hippocampal slices cultures. Rat hippocampal slice cultures were exposed to 60 min of oxygen glucose deprivation (OGD). Immediately after OGD slices were treated with varying doses of phenoxybenzamine. Then 21 h after OGD the slices were stained with propidium iodide and then visualized at 24 h. PBZ treatment from 0.1 μM–1 mM significantly reduced neuronal death from OGD in the CA1, CA3, and DG region of the hippocampus. *** *p* < 0.001, *n* = a minimum of seven slices; (**b**) non-OGD control; (**c**) OGD+phenoxybenzamine treated at 0.1 μM; (**d**) OGD + phenoxybenzamine treated at 10 μM; (**e**) OGD + phenoxybenzamine treated at 100 μM; (**f**) OGD + phenoxybenzamine treated at 1 mM; (**g**) OGD untreated control.

**Figure 2.**
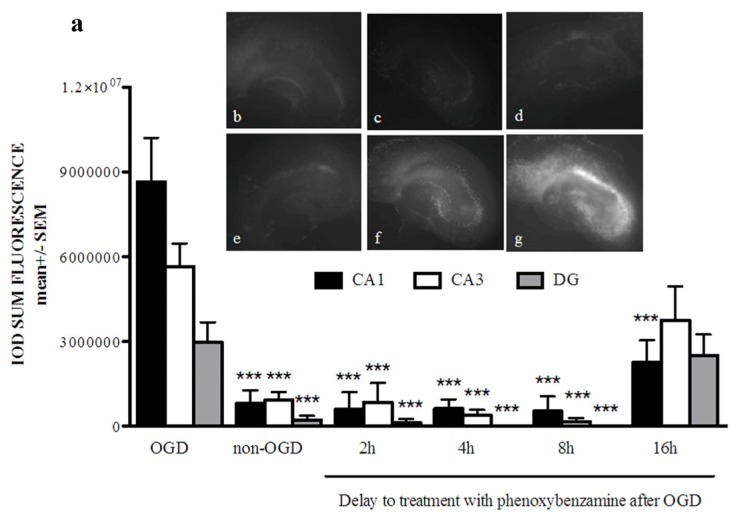
(**a**) Phenoxybenzamine (PBZ), delivered up to 16 h post injury, provides significant neuroprotection from oxygen glucose deprivation in rat hippocampal slice cultures. Rat hippocampal slice cultures were exposed to 60 min of OGD. At various time points after OGD, 100 μM PBZ was added to cultures. 21 h after OGD the slices were stained with propidium iodide and then visualized at 24 h. PBZ treatment at 100 μM significantly reduced neuronal death in the CA1, CA3, and DG region of the hippocampus when delivered at 2, 4, and 8 h post-OGD. Delivery of 100 μM PBZ at 16 h post-OGD significantly reduced neuronal death from OGD in the CA1 region of the hippocampus but did not reduce cell death in the CA3 or DG. *** *p* < 0.001, *n* = a minimum of seven slices; (**b**) non-OGD control; (**c**) OGD + phenoxybenzamine treated at 2 h post-OGD; (**d**) OGD + phenoxybenzamine treated at 4 h post-OGD; (**e**) OGD + phenoxybenzamine treated at 8 h post-OGD; (**f**) OGD + phenoxybenzamine treated at 16 h post-OGD; (**g**) OGD untreated control.

Our preliminary studies in the rat hippocampal slice culture (RHSC)-OGD model identified phenoxybenzamine as a potential neuroprotective candidate compound. We further tested phenoxybenzamine in the RHSC-OGD model by conducting a dose response study. Phenoxybenzamine preserved primary neurons within the CA1, CA3 and dentate gyrus and produced a robust neuroprotective effect over a broad dose range (0.1 μM–1 mM final media concentration) (Figure 1a). Neuroprotective compounds must be effective when administered at a clinically relevant time point, which in the case of stroke or traumatic brain injury can be hours after injury. Therefore, we examined the potential therapeutic window of phenoxybenzamine in the RHSC-OGD model. A middle dose (100 μM) was selected and added to the cultures at 2, 4, 8, or 16 h post-OGD. We found that phenoxybenzamine prevented neuronal death from OGD in all regions of the hippocampus when delivered at 2, 4, and 8 h post-OGD. When delivered at 16 h post-OGD, phenoxybenzamine prevented neuronal death only in the CA1 region of the hippocampus (Figure 2a). Taken together, these data demonstrated a neuroprotective role for phenoxybenzamine under OGD conditions.

### 2.2. Phenoxybenzamine Prevents Neurological Dysfunction that Occurs as a Result of Severe TBI

To test the effect of phenoxybenzamine in TBI, we utilized the rat lateral fluid percussion (LFP) injury model. The LFP model is one of the most widely used models of closed head injury and reproduces many of the conditions observed in humans following TBI [19]. In this model, a fluid pulse delivered to the intact dura produces a rotational torque and coup/contre coup typically observed with closed head injury [20,21]. In choosing our test dose, we extrapolated a relevant rat dose based on current FDA approved levels. In humans, phenoxybenzamine can be administered daily at doses up to 120 mg total [14,16,17,22]. Based on an average human weight of 70 kg, this represents 1.7 mg/kg. Therefore, we selected a slightly lower, single, i.v. dose of 1 mg/kg. This dose was then administered at the clinically relevant time point of 8 h after severe TBI. Therapeutic effectiveness was determined based on behavioral and cognitive outcomes.

**Figure 3.**
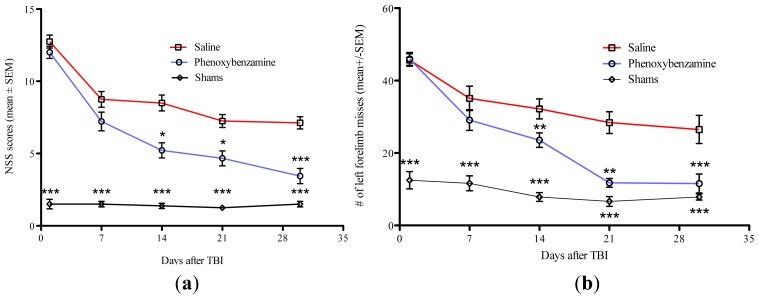
(**a**) Phenoxybenzamine, delivered 8 h after a severe traumatic brain injury (TBI) significantly reduced neurological impairment. PBZ at 1.0 mg/kg was delivered 8 h after a severe TBI. At 24 h and 7 days post-injury there was no significant difference in neurological impairment. Beginning on day 14 post-injury, significant differences in neurological severity scores were observed. This trend continued on day 21 and day 30 post-injury with significant differences observed between PBZ treated injured animals and saline treated injured animals. * *p* < 0.05, *** *p* < 0.001, *n* = a minimum of nine animals. (**b**) Phenoxybenzamine, delivered 8 hours after a severe TBI, significantly reduced foot fault errors. PBZ at 1.0 mg/kg was delivered 8 h after a severe TBI. At 24 h and 7 days post-injury there was no significant difference in foot fault errors. Beginning on day 14 post-injury, significant differences in foot fault errors was observed. This trend continued on day 21 and day 30 post-injury with significant differences observed between PBZ treated injured animals and saline treated injured animals. ** *p* < 0.01, *n* = 13 for saline treated TBI; *n* = 9 for phenoxybenzamine treated TBI animals; *n* = 8 for surgical shams.

Neurological severity score (NSS) and foot fault assessments were used to test the hypothesis that phenoxybenzamine treatment could improve behavioral outcomes. Animals were assessed 24 h after injury and on 7, 14, 21, and 30 days post-TBI. We found no significant differences in NSS or foot fault scoring between the saline treated controls and phenoxybenzamine treated animals at 24 h or 7 days after the TBI. These data confirm that all animals in both treatment groups experienced injuries of similar severity. However, phenoxybenzamine treated animals showed significant improvements in NSS and foot fault scoring on days 14, 21, and 30 (Figure 3a,b). It is worth noting that phenoxybenzamine treated rats had foot fault values that were not significantly different from uninjured control rats on days 21 and 30 of testing.

### 2.3. Phenoxybenzamine Reduces Cognitive Impairment Associated with Severe TBI

**Figure 4.**
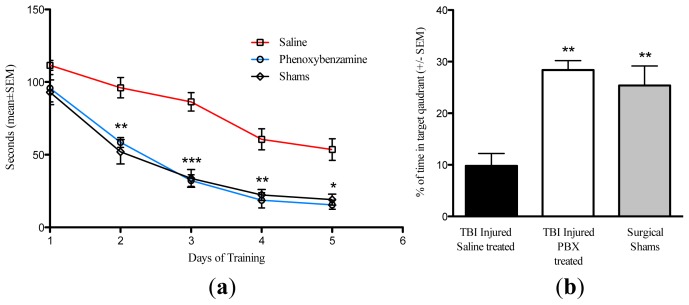
(**a**) Phenoxybenzamine, delivered 8 h after a severe TBI, significantly reduced learning impairment. Phenoxybenzamine at 1.0 mg/kg was delivered 8 hours after a severe TBI. Beginning 25 days post-TBI the animals were acclimated and trained in the Morris Water Maze. Animals were given four trials per day for two minutes per trial. Animals that failed to find the platform after two minutes were placed on the platform for 30 s to observe the visual cues. Beginning on day two of the training, the PBZ treated injured animals were significantly different from saline treated injured animals. This trend continued for the remainder of the training phase with significant differences between PBZ and saline treated animals present on days 2, 3, 4, and 5 of the training. For all five training days there were no significant differences between the un-injured shams and the PBZ treated animals. There was no significant difference in swim speeds for any of the animals in this study. * *p* < 0.05; ** *p* < 0.01; *** *p* < 0.001, *n* = 9 for saline treated TBI; *n* = 8 for phenoxybenzamine treated TBI animals; *n* = 8 for surgical shams. (**b**) Phenoxybenzamine, delivered 8 h after a severe TBI, significantly reduced memory impairment. Phenoxybenzamine at 1.0 mg/kg was delivered 8 h after a severe TBI. After the learning phase was completed, a probe trial was conducted to assess spatial memory function. The escape platform was removed and the animals were allowed to swim freely for 60 s. The amount of time spent searching in the target (platform) quadrant was recorded. During the probe trial the phenoxybenzamine treated animals displayed significantly greater spatial memory capacity when compared to the saline treated controls. The phenoxybenzamine treated TBI animals spent approximately 28% of available time searching the target quadrant for the escape platform compared to the 10% observed in saline treated TBI animals. As in the training phase, the phenoxybenzamine treated TBI injured animals did not differ from un-injured sham controls, which spent approximately 25% of their time searching the target quadrant. There was no significant difference in swim speeds for any of the animals in this study. ** *p* < 0.01, *n* = 9 for saline treated TBI; *n* = 8 for phenoxybenzamine treated TBI animals; *n* = 8 for surgical shams.

The Morris water maze (MWM) was used to assess cognitive function of rats beginning 25 days after injury. A single, i.v., administration of phenoxybenzamine at 8 h after TBI resulted in significant learning improvement on days 2–5 of the training phase (Figure 4a). Phenoxybenzamine treated animals were not significantly different from the un-injured, sham operated control animals on any of the training days. After the learning phase was completed, a probe trial was conducted to assess spatial memory function. During the probe trial the phenoxybenzamine treated animals displayed significantly greater spatial memory capacity compared to the saline treated controls (Figure 3b). The phenoxybenzamine treated TBI animals spent approximately 28% of available time searching the target quadrant for the removed escape platform. In contrast, saline treated controls spent approximately 10% of available time searching the target quadrant. As in the training phase, the phenoxybenzamine treated TBI injured animals did not differ from un-injured sham controls, which spent approximately 25% of their time searching the target quadrant (Figure 4b).

### 2.4. Phenoxybenzamine Reduces the Expression of Pro-Inflammatory Genes

In an effort to elucidate the possible mechanisms involved in phenoxybenzamine-mediated neuroprotection, we performed a gene array analysis of cortical tissue taken from animals at 32 h after the injury (8 h delay to treatment + 24 h after treatment). This time point was chosen in an effort to detect possible gene changes that would affect the development of secondary damage. The gene array screened for expression change in 24,0000 genes. From this initial screen, we identified a subset of 12 genes that appeared to exhibit significant changes and were potentially relevant to mechanisms of neuroprotection (Table 1). We further examined this subset of genes by quantitative rtPCR. Importantly, we detected a significant increase in the expression of the pro-inflammatory signaling proteins CCL2 (11 fold, *p* = 0.004), IL1β (4.6 fold, *p* = 0.005) and MyD88 (3 fold, *p* = 0.0001) following severe TBI (Table 1). In contrast, rats treated with phenoxybenzamine after severe TBI showed no significant increase in the expression of these proinflammatory genes. These data suggest that phenoxybenzamine may mediate neuroprotection by modulating the neuroinflammatory response. TBI also induced significant changes in cellular retinol binding protein 2 (Rbp2) and corticotrophin releasing hormone (CRH). However, similar changes to the expression of these genes were seen in both saline and methamphetamine treated groups. Finally, TBI induced a slight but statistically significant increase in the expression of the calcium permeable AMPA receptor (GRIA4). In contrast, methamphetamine treatment resulted in no significant increase in GRIA4 expression.

### 2.5. Discussion

We have demonstrated that a single i.v. administration of 1.0 mg/kg of phenoxybenzamine, delivered 8 h after TBI, significantly reduced both behavioral and cognitive impairment. This represents a novel finding as there are no previous studies indicating that phenoxybenzamine exerts any type neuroprotective effects. Currently, phenoxybenzamine is used to treat hypertension and excessive sweating associated with adrenal tumors (pheochromocytoma). Interestingly, phenoxybenzamine does not appear to decrease global cerebral blood flow [23]. It does, however, appear to re-distribute flow among certain regions of the brain [23]. Buchweitz and Weiss [23] performed studies in which they found phenoxybenzamine reduced blood flow in certain cortical regions and the hippocampus, but increased blood flow in the pons and substantia nigra. Mechanistically, phenoxybenzamine acts as a potent α-1 adrenergic antagonist (with secondary α-2 antagonism) and thus blocks the effects of epinephrine and norepinephrine [16,24]. There is evidence that suggests blocking the effects of epinephrine and norepinephrine may provide significant benefits to TBI patients. Severe TBI increases the activity of the sympathetic nervous system resulting in the excessive release of epinephrine and norepinephrine [25]. Previous research indicates a direct correlation between the severity of TBI, plasma epinephrine and norepinephrine levels, and recovery rates [25]. Patients remaining in a persistent coma have epinephrine and norepinephrine plasma levels several-fold higher than controls. Furthermore, these catecholamine levels remain elevated for the duration of the comatose state. Conversely, TBI patients with initial catecholamine levels that are mildly elevated have been found to consistently improve to a Glasgow Comma Scale (GCS) value greater than 11 at one week post-TBI. In patients with multisystem trauma and TBI, plasma norepinephrine levels at 48 h post injury were predictive of the GCS at one week [26,27]. Plasma norepinephrine also correlated with patient survival, the number of ventilator days, and the length of hospital stay [24,26,27].

In terms of cognitive function, the increased presence of norepinephrine in TBI has been associated with poor neuropsychological outcome. Kobori *et al*. [28], found that the administration of the α-1 antagonist Prazosin, at 14 days after a TBI, significantly improved working memory in rats [28]. Kobori *et al*. [28] went on to elucidate a proposed mechanism that linked the impaired working memory with TBI mediated increase in the presence of α-1 in the medial pre-frontal cortex. The increased α-1 was linked to a CREB mediated increase in α-1 receptor gene transcription through binding of phospho-CREB to CRE sites on the α-1A receptor suggesting a positive feedback effect.

Based on these studies, the neuroprotective effect of phenoxybenzamine may be a direct result of the α-1 and α-2 antagonism that blocks the secondary effects of norepinephrine signaling in the brain. The alpha 1 adrenergic receptor is coupled to a heterotrimeric G protein, G_q_, which activates phospholipase C (PLC) [29]. PLC produces an increase in IP_3_ and calcium, which, in turn, activates protein kinase C (PKC) [30,31]. Previous studies in TBI have demonstrated that PKC is rapidly elevated as a result of the injury [30]. Another potential mechanism of neuroprotection associated with phenoxybenamine may be a reduction in calmodulin(CaM)/CaMKII activity. Phenoxybenzamine is also a potent inhibitor of CaM/CaMKII activity [29]. Under basal conditions CaMKII is a major mediator of glutamate signaling, however, under acute injury conditions, CaM/CaMKII interacts with the NR2B subunit of NMDA receptors leading to excitotoxic death [32]. There is evidence that CaM/CaMKII increases the trafficking of AMPA receptors to the cell surface leading to greater excitotoxic death during acute injury [32]. Supporting a neurodestructive role for CaMKII, Zhang *et al.* [33] found that TBI increased the expression of CaMKIIδ. Pre-treating rats with a CaMKIIδ inhibitor before TBI resulted in a significant decrease in lesion volume and a significant increase in neuromotor function [33]. Zhang *et al*. [33] went on to elucidate a mechanism in which CaMKIIδ actively promotes apoptosis in neurons by increasing the pro-apoptotic protein BAX and subsequent caspase 3 activation [33].

From gene array studies we found that phenoxybenzamine appears to block critical gene changes that occur after TBI. Genes involved in inflammation such as CCl2, IL-1β, and MyD88 were all significantly elevated in TBI animals, but phenoxybenzamine treated animals did not differ from uninjured control rats. This is a crucial finding because inflammation contributes to the formation of edema, a loss of neurons, and negatively affects patient recovery. A key component of brain inflammation is the recruitment of neutrophils and monocytes, which are toxic to neurons [34–38]. Recruitment of monocytes into the brain is primarily controlled by monocyte chemotactic protein 1 (MCP-1) also known as CCL2, which is expressed by astrocytes, macrophages, and reactive microglia [36,39]. After TBI, CCL2 actively recruits monocytes to areas of brain damage leading to inflammation, edema and neuronal damage [39]. Rhodes *et al*. [40], found a rapid increase in CCL2 in human spinal fluid following severe TBI. Furthermore, CCL2 levels remained significantly elevated for up to 10 days post-injury [34]. Semple *et al*. [34] reported that elevated levels of CCL2 were detected in the serum of patients who died after TBI.

Epithelial cells actively synthesize CCL2 in response to the proinflammatory cytokine, interleukin-1β (IL-1β) [41,42]. From our gene array analysis, we found that saline treated animals had a significant increase in IL-1β after TBI whereas phenoxybenzamine treated animals showed no significant increase. In terms of TBI, IL-1β is the primary activator of microglia and is directly responsible for inducing inflammation [6,8,43]. IL-1β further contributes to immunoexcitoxicty by enhancing the sensitivity of NMDA receptors and tumor necrosis factor alpha (TNF-α) leading to increased brain inflammation and excitotoxicity [8,44–46].

Phenoxybenzamine may further reduce post-traumatic inflammation by reducing expression of myeloid differentiation primary response protein 88 (Myd88). Myd88 is a key adaptor protein involved in Toll-like receptor and pro-inflammatory cytokine signal transduction [47,48]. In saline treated TBI animals, Myd88 was significantly up-regulated over uninjured controls. However, MyD88 expression levels were equivalent to uninjured controls in phenoxybenzamine treated rats after TBI. Mechanistically, Myd88 is a key adaptor protein for Toll-like receptors, cytokines, and nuclear factor kappaB (NF-κB) [47,48]. Previous research suggests activation of Toll-like receptors results in the recruitment of Myd88 and the subsequent activation of NF-κB, which in turn, induces the rapid expression of pro-inflammatory molecules including tumor necrosis factor a (TNF-α), IL-1β, interleukin-6 (IL-6), and intracellular adhesion molecule-1 (ICAM-1) leading to a pro-inflammatory response [47–50]. These data strongly suggest that phenoxybenzamine is a valid candidate for further investigation as a potential neuroprotective agent and treatment for TBI.

## 3. Experimental Section

### 3.1. Rat Organotypic Hippocampal Slice Cultures

All experimental animal procedures were approved by the University of Montana Institutional Animal Care and Use Committee in accordance with National Institutes of Health guide for the care and use of Laboratory animals (NIH Publications No. 8023). Hippocampal slice cultures were prepared from the brains of 7-day-old Sprague-Dawley rat pups as previously described [51]. After 7 days in culture, slices were exposed to oxygen-glucose deprivation. A glucose free balanced salt solution (BSS) composed of 120 mM NaCl (EMD Science, Gibbstown, NJ, USA), 5 mM KCl (Sigma Aldrich, St. Louis, MO, USA), 1.25 mM NaH_2_PO_4_ (EMD Science, Gibbstown, NJ, USA), 2 mM MgSO_4_ (Sigma Aldrich, St. Louis, MO, USA), 2 mM CaCl_2_ (Sigma Aldrich, St. Louis, MO, USA), 25 mM NaHCO_3_ (Sigma Aldrich, St. Louis, MO, USA), 20 mM HEPES (EMD Science, Gibbstown, NJ, USA), 25 mM sucrose (Sigma Aldrich, St. Louis, MO, USA); pH 7.3 was bubbled for 1 h with 5% CO_2_/95% N_2_ at 10 L/h. Slices were washed 6 times in deoxygenated SBSS to remove residual glucose, transferred into deoxygenated SBSS and placed in a 37 °C chamber with a ProOx 110 oxygen feedback sensor (BioSpherix, Lacona, NY, USA) that maintained gas levels at 0.1% O_2_, 5% CO_2_, 94.4% Nitrogen for 60 min. After OGD, the slices were immediately transferred back into pre-warmed Neurobasal media containing B27 without anti-oxidants (Life technologies Carlsbad, CA, USA) under normal oxygen conditions. Slices treated with phenoxybenzamine (Sigma Aldrich, St. Louis, MO, USA) in the dose-response study were placed in pre-warmed Neurobasal media containing 0.1 μM–1 mM phenoxybenzamine immediately after OGD. For the time course studies, 100 μM phenoxybenzamine was added to Neurobasal media at 2, 4, 8, or 16 h after OGD. Neuronal damage was determined by staining slices with propidium iodide (PI) (Molecular Probes, Eugene, OR, USA) and quantifying the relative fluorescence intensity (excitation 540/emission 630) using ImagePro Plus software (Media Cybernetics, Silver Springs, MD, USA). PI was added to the media at a concentration of 2 μM [52], 4 h prior to OGD. Images were taken of the hippocampal slices prior to OGD to establish baseline fluorescence. After OGD, slices were placed in normal media containing 2 μM PI and imaged again at 24 h post-OGD using fluorescence optics with an Olympus IMT-2 microscope and a Hamamatsu camera. The total fluorescent intensity in each slice was determined using ImagePro Plus software (Media Cybernetics, Silver Springs, MD, USA) and all values were expressed as an integrated optical density (IOD) which calculates the area multiplied by the pixel number and intensity to give a total fluorescent staining intensity that measures the amount of cell death.

### 3.2. TBI Procedure

Male Wistar rats (350–500 g) were obtained from Charles River Laboratories (Wilmington, MA, USA) and housed with a 12-h light/dark cycle and ad libitum access to food and water. Severe TBI was induced using the LFP procedure as previously described [18]. Briefly, a 5 mm craniotomy was made over the right hemisphere equidistant between the lambda and the bregma. Animals were given a fluid pulse to the brain at 1.9–2.3 ATM of pressure with a 20 ms duration using an FP 302 LFP machine (AmScien Instruments, Richmond, VA, USA). All animals experienced apnea and were manually ventilated on 0.5 liters O_2_/min until normal breathing occurred. Throughout the procedure and for 3 h post-injury, body temperature, heart rate, and spO_2_ levels were monitored and recorded. Animals had an average righting time of 24 min and an average mortality rate of 25% was observed. Mild to moderate TBI injuries (NSS < 9) that were excluded from the study was 14%. At 8 h post-TBI, phenoxybenzamine hydrochloride (Sigma Aldrich, St. Louis, MO, USA) at 1 mg/kg dissolved into 200 μL of sterile saline was injected into the tail vein of randomly selected rats using a 25-gauge needle. Saline treated animals underwent the same tail vein injection procedure but received 200 μL of saline. Sham operated animals received a craniotomy but did not receive a TBI. Post surgery, the animals were monitored twice daily for one week. Each day of the first week following TBI, all animals were given 10 mL pre-warmed supplemental saline given subcutaneously, and 100 g of AD Veterinary diet (to stimulate eating and minimize weight loss). All animals were weighed once daily for two weeks post TBI.

### 3.3. Neurological Severity Scoring

Neurological severity scoring (NSS) and foot fault assessments were performed as previously described [10,18]. Assessements were conducted on days 1, 7, 14, 21, and 30 by a blinded observer. Scores ranged from 0–16 with 16 indicating maximal impairment. Scoring criteria for a severe TBI was an NSS in the range of 10–16. Animals scoring an NSS of 9 or less on day 1 post injury were identified as having a moderate/mild injury and were excluded. Animals that scored 10 or greater on their NSS were randomly distributed into saline or phenoxybenzamine treated groups. For this study 13 animals were placed in the saline group with a mean day 1 NSS of 12.7 and nine animals were placed in the phenoxybenzamine group with a mean day 1 NSS of 12.0.

### 3.4. Foot Faults Assessments

Foot fault assessments were conducted as previously described [10,18]. Briefly, rats were set on an elevated grid. With each weight-bearing step, the paw may fall or slip off the wire grid. Each time the left forelimb (affected by damage to the right hemisphere) missed a placement on the wire rack it was recorded as a foot fault. The total number of steps that the rat used to cross the grid was counted, and the total numbers of foot faults for the left forelimb was recorded. The total number of left forelimb misses out of 100 forelimb steps was reported.

### 3.5. Assessment of Cognitive Function

The Morris water maze (MWM) was used to assess the impact of phenoxybenzamine on cognitive function (learning and memory) following TBI. The assessment procedure was performed as previously published [18]. Pre-acclimation began on day 24 post-TBI. The training phase began on day 25 post-injury, and the probe trial was conducted on day 30 post-injury. The water temperature was maintained at a constant 19 degrees Celsius with the clear plexiglas escape platform 2 cm below the water level. All data was recorded and analyzed using Anymaze software connected to a Logitech camera. All data sets were analyzed by a blinded researcher. There were no significant differences in swim speeds between any of the animals tested.

### 3.6. RNA Isolation/Qrt-PCR

Three biological replicates were used for each experimental group and each replicate was run in triplicate. Total RNA was isolated from the rat ipsilateral cortex utilizing Trizol LS (Life technologies Carlsbad, CA, USA) according to the manufacturer’s protocol. To remove any contaminating chemicals that could affect downstream analysis, RNA was further purified using the RNeasy MinElute Cleanup Kit (Qiagen, Valencia, CA, USA) according to the manufacturer’s protocol. RNA was quantified using a Nano Drop spectrophotometer (Cole-Parmer, Vernon Hills, IL, USA. RNA was considered acceptable if the 260/280 and 260/230 ratios were 2.0 or greater. A 1 μg aliquot of total RNA was reverse transcribed to cDNA using Qiagen’s RT2 First Strand Kit (Qiagen, Valencia, CA, USA). One half of the cDNA reaction was mixed with 675 μL of Qiagen’s 2X RT SYBR Green Mastermix and 624 μL H_2_O. This was then added to a custom array panel (SA Biosciences, Valencia, CA, USA) and cycled according to the manufacturer’s suggested qPCR protocol on a iQ5 thermocycler (Bio-Rad, Hercules, CA, USA). Values were normalized for GAPDH. For data analysis, the ΔΔ*C*t method was used with the aid of the manufacturer’s online software suite RT2 Profiler PCR Array Data Analysis version 3.5 (SA Biosciences, Valencia, CA, USA). Fold-changes were calculated and results compared to normal controls. Please note: (2^−ΔΔ^*^C^*^t^) is the normalized gene expression (2^−Δ^*^C^*^t^) in the test sample divided the normalized gene expression (2^−Δ^*^C^*^t^) in the Control Sample. Fold-Regulation represents fold-change results in a biologically meaningful way. Fold-change values greater than one indicate a positive- or an up-regulation, and the fold-regulation is equal to the fold-change. Controls for this experiment underwent the trephination surgery but did not receive a TBI.

### 3.7. Statistical Analysis

All data was analyzed using Prizm software (GraphPad, La Jolla, CA, USA). To determine Gaussian (normal) distribution a Kolmogorof-Smirnov test was performed on all data sets. Analysis on data sets with more than two groups was done using one-way ANOVA with Tukey’s *post-hoc* to determine statistical significance between groups. A *p* < 0.05 or less was considered significant.

## 4. Conclusions

Clinically, phenoxybenzamine is FDA approved and currently used to treat hypertension and sweating associated with pheochromocytoma, a condition in which the adrenal glands secrete high levels of noradrenaline and adrenaline. As a therapeutic, the side effects of phenoxybenzamine are minimal: stuffy nose, mild drowsiness, blurred vision, and upset stomach. As a treatment for TBI it possesses several advantages. It can be delivered intravenously, it efficiently crosses the blood brain barrier, it produces mild side effects, and it significantly reduces neurological and cognitive dysfunction when delivered 8 h after a severe TBI. Taken together, it is our opinion that phenoxybenzamine warrants further investigation as a potential neuroprotective agent for TBI.

## Figures and Tables

**Table 1.** Gene Expression Analysis.

Genes	Saline		Phenoxybenzamine	

	Fold Change	*p*-value	Fold Change	*p*-value
*CCL2*	11.12	0.004	3.34	0.92
*CXCL12*	1.58	0.04	1.02	0.55
*Fos*	1.46	0.23	−1.64	0.08
*IL1β*	4.58	0.005	1.53	0.71
*Gadd45g*	2.22	0.69	1.02	0.16
*Myd88*	3.03	0.0001	1.27	0.20
*Pdgfα*	1.21	0.10	−1.28	0.09
*NTS*	1.12	0.60	1.23	0.19
*Rbp2*	3.96	0.00008	1.58	0.03
*CRH*	−1.66	0.002	−1.56	0.003
*GRIA4*	1.47	0.005	−1.14	0.50
*GRIA2*	1.11	0.42	−1.17	0.19
